# First‐in‐human clinical trial of allogeneic, platelet‐derived extracellular vesicles as a potential therapeutic for delayed wound healing

**DOI:** 10.1002/jev2.12332

**Published:** 2023-06-23

**Authors:** Jancy Johnson, Sam Q. K. Law, Mozhgan Shojaee, Alex S. Hall, Sadman Bhuiyan, Melissa B. L. Lim, Anabel Silva, Karmen J. W. Kong, Melanie Schoppet, Chantelle Blyth, Hansi N. Ranasinghe, Nenad Sejic, Mun Joo Chuei, Owen C. Tatford, Anna Cifuentes‐Rius, Patrick F. James, Angus Tester, Ian Dixon, Gregor Lichtfuss

**Affiliations:** ^1^ Exopharm Ltd Melbourne VIC Australia; ^2^ Department of Biochemistry and Pharmacology University of Melbourne Parkville VIC Australia

**Keywords:** extracellular vesicles, first‐in‐human platelet EV therapy, platelet EVs, wound healing

## Abstract

The release of growth factors, cytokines and extracellular matrix modifiers by activated platelets is an important step in the process of healthy wound healing. Extracellular vesicles (EVs) released by activated platelets carry this bioactive cargo in an enriched form, and may therefore represent a potential therapeutic for the treatment of delayed wound healing, such as chronic wounds. While EVs show great promise in regenerative medicine, their production at clinical scale remains a critical challenge and their tolerability in humans is still to be fully established. In this work, we demonstrate that Ligand‐based Exosome Affinity Purification (LEAP) chromatography can successfully isolate platelet EVs (pEVs) of clinical grade from activated platelets, which retain the regenerative properties of the parent cell. LEAP‐isolated pEVs display the expected biophysical features of EV populations and transport essential proteins in wound healing processes, including insulin growth factor (IGF) and transforming growth factor beta (TGF‐ß). In vitro studies show that pEVs induce proliferation and migration of dermal fibroblasts and increase dermal endothelial cells' angiogenic potential, demonstrating their wound healing potential. pEV treatment activates the ERK and Akt signalling pathways within recipient cells. In a first‐in‐human, double‐blind, placebo‐controlled, phase I clinical trial of healthy volunteer adults, designed primarily to assess safety in the context of wound healing, we demonstrate that injections of LEAP‐purified pEVs in formulation buffer are safe and well tolerated (Plexoval II study, ACTRN12620000944932). As a secondary objective, biological activity in the context of wound healing rate was assessed. In this cohort of healthy participants, in which the wound bed would not be expected to be deficient in the bioactive cargo that pEVs carry, all wounds healed rapidly and completely and no difference in time to wound closure of the treated and untreated wounds was observed at the single dose tested. The outcomes of this study evidence that pEVs manufactured through the LEAP process can be injected safely in humans as a potential wound healing treatment, and warrant further study in clinical trials designed expressly to assess therapeutic efficacy in patients with delayed or disrupted wound healing.

## 1 INTRODUCTION

Delayed wound healing represents an area of significant unmet medical need. Chronic wounds affect 1%–2% of the population in developed world countries, costing more than US$25 billion annually in healthcare (Järbrink et al., 2016). Individuals with comorbidities including vascular disease and diabetes are at increased risk of chronic wound onset.

Chronic wounds arise when wound healing processes become disrupted. Normal, healthy wound healing consists of four sequential, overlapping phases: haemostasis; inflammation; cell proliferation; and re‐modelling culminating in wound closure. Chronic wounds, however, typically enter a persistent inflammatory state and fail to transition through the cell proliferation and remodelling phases of healing (Falanga et al., 2022; Zhao et al., 2016).

Platelets are the first responders to injury within the body and have been shown to possess a remarkable ability to induce tissue growth and repair (Eisinger et al., 2018). Therefore, platelets and their derivatives, including platelet‐rich plasma (PRP) and platelet lysate, have been investigated to reinitiate wound healing. However, reports of efficacy in clinical trials have been variable. One likely source of variability may be the absence of a clear gold standard protocol for platelet activation and PRP production (Cardeñosa & Domínguez‐Maldonado, 2017; Marck et al., 2016; Martinez‐Zapata et al., 2012). Therefore, platelet numbers and growth factor content of PRP treatments may differ from dose to dose. In addition, blood plasma can contain high levels of anti‐angiogenic factors, which may counteract the platelets’ beneficial pro‐angiogenic effects (Etulain, 2018).

Upon activation, platelets release intracellular stores of growth factors, cytokines, and extracellular matrix modulators (Torreggiani et al., 2014). These factors can be released as free proteins but are also released within extracellular vesicles (EVs) in an enriched form compared to the parent cell (Torreggiani et al., 2014). EVs are nanometre‐scale lipid membrane‐bound particles that deliver proteins, lipids, and nucleic acids from parent cells to recipient cells (Tao et al., 2017). EVs are released by almost every cell type in the human body, including platelets, as natural mediators of cell‐to‐cell signalling.

Several studies have demonstrated the capability of platelet EVs (pEVs) to improve cellular processes essential to wound healing, including proliferation, angiogenesis, and immunomodulation (Guo et al., 2017; Mause et al., 2010). pEVs have been shown to rescue aberrant fibroblast and endothelial cell behaviour and restart the healing process (Guo et al., 2017) and have been shown to be the prime mediators of efficacy in platelet treatments (Varon & Shai, 2015). Furthermore, EVs can be highly stable in blood and home to damaged tissue (Simeone et al., 2020). However, current EV isolation and purification methods rely on tedious, slow, and non‐scalable processes (Colao et al., 2018; Wiklander et al., 2019), limiting the applicability of pEVs as wound healing medicines in the clinic.

In this study, we provide the first evidence for the safety and therapeutic utility of clinical‐grade pEVs to humans. We demonstrated that pEVs isolated by a novel chromatography‐based process, Ligand‐based Exosome Affinity Purification (LEAP), retain regenerative properties of their parent cell type. LEAP‐isolated pEVs induced healing‐associated proliferation, migration, and angiogenic behaviours in fibroblast and dermal endothelial cells. Proteomic analysis of the isolated pEVs revealed the presence of multiple growth factors, and we confirmed that cellular response to pEVs was mediated at least in part by their activation of the ERK and Akt signalling pathways. As a step toward developing pEVs as a potential therapeutic for chronic wounds, LEAP‐isolated pEVs were formulated as a clinical‐grade, allogeneic, candidate therapeutic product, and assessed in a randomised placebo‐controlled phase I clinical trial for wound healing in healthy volunteers. No significant adverse effects were observed. These results show that pEVs manufactured through the LEAP isolation process warrant clinical investigation as a therapeutic for delayed wound healing, such as chronic wounds.

## 2 METHODS

### 2.1 Ethics statement and collection of platelet packs

Human research ethics approval for the use of human platelets was obtained by Australian Red Cross LifeBlood. All donors were approved for collection by Australian Red Cross LifeBlood, also our clinical‐grade platelet provider.

The pooled platelet packs were prepared by combining the buffy coats of four ABO identical whole blood donations and resuspending with platelet additive solution (PAS) (MacoPharma, Mouvaux, France) to 70% of the final volume (i.e., 30% buffy coat). Each pooled platelet pack was prepared to a platelet count of >240 × 10^9^ platelets per pack and was leukocyte‐depleted and gamma‐irradiated. Platelet packs were also screened for ABO and RhD groups, red blood antibody, bacteria contamination, and transfusion‐transmissible viruses (HIV, HBV, HCV, HTLV) prior to release for use. Each pack contained a volume of 380 ± 10 mL.

### 2.2 Purification of platelet‐derived extracellular vesicles (pEVs) by ligand‐based exosome affinity purification (LEAP)

Each pooled pack contained platelets suspended in 30% plasma/70% PAS, as previously described (Johnson et al., 2018). The platelets were captured on a depth filter media to remove the plasma content from the packs and then washed with 300 mL of PAS. Platelets were then activated using Exopharm's in‐house agonist‐free activation method. Platelet releasate was collected with a further wash with 500 mL of PAS. The platelet releasate containing pEVs was then filtered using a 0.45 μm filter to remove any remaining platelet debris.

Further purification of pEVs from the releasate was performed using Ligand‐based Exosome Affinity Purification (LEAP) column chromatography, as previously described (Law et al., 2021). Briefly, the filtered releaseate was loaded onto a chromatography column packed with a LEAP resin. After washing the column to remove contaminants, pEVs were eluted from the column by a buffer change. The eluted EVs were buffer exchanged for a total of seven diavolumes into a proprietary formulation buffer using a centrifugal filter (Amicon Ultra‐15, 30 kDa MWCO regenerated cellulose, Merck Germany), with each spin cycle of 10–15 min at 2000 rpm to reach the desired buffer exchange diavolume. LEAP‐purified pEVs were 0.45 μm filtered and snap‐frozen at −80°C until further use. Before release, the product was tested for sterility (BD BACTEC anaerobic and aerobic bottles) and endotoxin.

### 2.3 Determination of protein concentration of LEAP‐purified pEVs

To determine the amount of protein within each pEV sample, a bicinchoninic acid (BCA) assay was performed following the manufacturer's protocol (Thermo Scientific, Scoresby, Victoria, Australia). Briefly, 20 μL of the sample was homogenized with 60 μL of radioimmunoprecipitation assay buffer (lysis buffer) and incubated at room temperature for 5 min. To each well of a 96‐well plate, 25 μL of lysed sample and 200 μL of working reagent were added, then incubated for 30 min at 37°C before being analysed on the spectrophotometer (Clariostar, BMG Labtech, Germany) at 562 nm.

### 2.4 Nanoparticle size analysis

To measure the size and concentration of particles in each pEV sample, a microfluidic resistive pulse sensing device (Spectradyne nCS1, CA, USA) was used. For nanoparticle size analysis, the isolated pEV sample was diluted to 1:20 with PBST, and 5 μL was aliquoted into a TS‐400 cartridge (measurement range 50–400 nm). Spectradyne acquisition time was set at 10 s. The first acquisition of each sample was excluded from processing. Datasets for each sample were collected until the apparent error of the particle analysis was less than 0.5% based on the default processing conditions.

Diameter correction factor was determined by the analysis of three size standards. To cover the expected pEV size range, National Institute of Standards and Technology traceable particle size monodisperse polystyrene beads of 92.8, 150, and 208 nm size were used as a mixture. The standard mix was run on the same chipset as the samples analysed. Post‐processing of the data was conducted, which included the subtraction of background particles (20 nm filtered PBST). Default size and transit time filters were added to exclude data points from the diluent buffer and electronic noise. The area under the curve calculations was conducted (Viewer software, Spectradyne, CA, USA) to determine the concentration of particles within selected size ranges.

### 2.5 Cryo‐transmission electron microscopy

To visualize LEAP‐purified pEVs, cryo‐transmission electron microscopy (TEM) was performed. The vesicles were imaged on a Talos L120C (ThermoFisher) transmission electron microscope, equipped with a LaB6 cathode filament at 120 kV (Melbourne Advanced Microscope Facility, Bio21 Institute, Australia). Cryogenic sample preparation was performed on a Vitrobot Mach IV (ThermoFisher) using lacey and holey carbon grids. For each grid, 4 μL of the sample was blotted for 7 s and plunge‐frozen in liquid ethane before imaging.

### 2.6 Western blot

Western blots were used to detect characteristic EV proteins within LEAP‐purified pEVs. Protein extracts from donor platelets and purified EVs were generated with radioimmunoprecipitation assay (RIPA) buffer (Abcam) with a complete protease inhibitor cocktail (Roche). Following sodium dodecyl sulphate‐polyacrylamide gel electrophoresis (SDS‐PAGE), the separated protein was transferred onto polyvinylidene fluoride membranes. The membranes were blocked with 5% non‐fat milk (Biotium) or 3% bovine serum albumin (Sigma) where appropriate, in 20 mM Tris‐HCl (pH 7.5), 500 mM NaCl plus 0.05% Tween (TBS‐T) and immunoblotted at 4°C overnight with primary antibodies: Syntenin (rabbit monoclonal IgG; Cat. no. ab133267, Abcam), CD9 (mouse monoclonal IgG; Cat. no. 312102, Biolegend), CD63 (mouse monoclonal IgG; Cat. no. sc‐5725, Santa Cruz), Calnexin (rabbit polyclonal IgG, Cat. no. ab22595, Abcam). The membranes were extensively washed in TBS‐T and incubated with peroxidase‐conjugated anti‐rabbit IgG antibody (Cat. no. ab6721, Abcam; or Cat. no. 7074S, Cell Signalling Technology) or anti‐mouse IgG antibody (Cat. no. ab6728, Abcam), for 1 h at room temperature. After washing with TBS‐T, the reaction was visualized using ECL detection reagents (SuperSignal™ West Pico PLUS Chemiluminescent Substrate, Thermo Scientific) with a G:Box F3 gel doc system (Syngene).

### 2.7 Proteomic analysis

Mass spectrometry proteomic analysis was conducted on LEAP‐purified pEVs to identify the proteins present. A modified Folch extraction was performed on the samples to separate peptides and other water‐soluble components from the lipid solution. The aqueous solution was then injected into the column for analysis on a U3000 HPLC in‐line (Thermo Fisher, San Jose, CA, USA) with an Orbitrap Lumos Fusion (Mass Spectrometry and Proteomics Facility, Bio21 Institute, Australia). The data from each analysis were searched using the Protein Discoverer 2.4 software platform (Thermo Fisher, San Jose, CA, USA). The search parameters were 5 ppm tolerance for the precursor ion and 0.02 Da tolerance for the product ion. Enzymatic cleavage with trypsin with two allowable missed cleavages. Fixed modification was Carbamidomethylation on Cys; variable modifications were oxidation on Met and deamidation on Asn & Gln. The search database was Swissport, all species. The search results were then manually curated.

### 2.8 Cell association assay

To demonstrate that pEVs purified by LEAP technology were intact and biologically active, their uptake by recipient cells was monitored by fluorescence microscopy using the lipophilic dye, Exoria (Tertel et al., 2022). LEAP‐isolated pEVs (approximately 10^11^ particles/mL) were labelled with a final concentration of 2 μM Exoria and incubated at 37°C for 1 h (Law et al., 2021). Free dye was removed with Zeba spin columns, 40K MWCO, as per the manufacturer's instructions (Thermo Fisher, San Jose, CA, USA). Normal human dermal fibroblasts (NHDFs; ATCC) were grown on 96 well glass bottom microplates (Greiner Bio‐One, Kremsmünster, Austria). 20 μL of Exoria‐labelled pEVs or Exoria in PBS (control) were added onto cells and incubated at 37°C for 2 h. Supernatants were removed and the cells were washed three times with PBS. Cells were counter‐stained with 10 μM Hoechst 33342 (Thermo Fisher, San Jose, CA, USA) at room temperature in the dark for 10 min. Cells were washed three times with PBS, then 50 μL of Fixation buffer (Biolegend, San Diego, CA, USA) was added, and cells were incubated at room temperature in the dark for 15 min. Cells were washed three times with PBS, then left in PBS for imaging with a confocal microscope (Nikon A1R, inverted Eclipse Ti‐E model. Images analysed using Image J) (Tertel et al., 2022).

### 2.9 Proliferation assay

A real‐time proliferation assay was performed to observe the effect of LEAP‐purified pEVs on fibroblasts. NHDFs at 5000 cells per well were seeded on 96‐well E‐plates (Agilent Technologies, USA) in a complete growth medium containing 2% v/v serum, then placed in an xCELLigence RTCA eSight instrument (Agilent Technologies, USA) and their growth monitored overnight. Cells were washed, media was replaced with basal media containing 0.1% v/v serum, and their growth was monitored overnight. Cells were then treated with 15 μg/mL of LEAP‐isolated pEVs (h 0), incubated at 37°C with 5% CO_2_, and growth was monitored for 70 h in the xCELLigence RTCA eSight instrument.

### 2.10 Migration assay

A scratch assay was performed to determine if LEAP‐purified pEVs influenced cell migration. Briefly, 12,000 NHDFs per well were seeded on a 96‐well plate. When a confluent monolayer was formed, a uniform scratch was made in each well by AccuWound 96 tool (ACEA Biosciences, USA). The wells were washed to remove cell debris, and pEV treatments (15 μg/mL) were added to the appropriate well. After 24 h of treatment, the wells were stained with Hoechst nuclear stain, imaged, and the number of migrated cells was quantified using ImageJ.

### 2.11 Angiogenesis assay

To investigate whether LEAP‐purified pEVs induce angiogenesis, tube formation by human dermal endothelial cells (HDMECs, PromoCell, Germany) was observed. Briefly, 7500 cells per well were seeded on 96‐well plates (Corning, USA) and allowed to adhere for 2 h. Cells were then treated with 15 μg/mL of pEVs. Tube formation capacity was assessed by observing the vessel‐like structures formed over 120 h after treating the cells using a light microscope.

### 2.12 ERK and Akt pathway activation analysis

To verify whether LEAP‐purified pEVs treatment induced ERK or Akt phosphorylation, AlphaLISA assays were performed according to the manufacturer's recommendations to quantify the amount of total and phosphorylated ERK and Akt proteins present in dermal fibroblasts after treatment with pEVs. Briefly, normal human dermal fibroblasts were either untreated or treated with pEVs (15 μg/mL). After 1, 2.5, 5, 15, 30, and 60 min of treatment, cells were lysed, and resulting lysates were used as analytes in the AlphaLISA assays run in 384‐well plates (OptiPlate, PerkinElmer). Acceptor beads conjugated with biotinylated antibodies were added to the samples and allowed to incubate in the dark for 60 min at room temperature. Streptavidin‐coated donor beads were added, followed by a secondary incubation in the dark for 60 min. The AlphaLISA signal was read on the Clariostar spectrophotometer at an excitation wavelength of 680 nm.

### 2.13 Phase I clinical trial design

In a first‐in‐human study, a phase I clinical trial in which healthy volunteers were enrolled to assess the safety of human allogeneic pEVs manufactured through the LEAP protocol was conducted (Plexoval II study, ACTRN12620000944932). LEAP isolation of clinical‐grade pEVs was conducted under GMP‐like protocols, performed in an ISO5 BioSpherix GMP Isolator Unit housed in an ISO8 certified clean room. Prior to release for human use, quality control in the form of sterility testing, endotoxin testing, and particle and protein concentration analysis, was performed.

Plexoval II was a prospective, randomised, double‐blind, placebo‐controlled, single dose, single site clinical trial conducted in Melbourne, Australia. The study was conducted following Good Clinical Practice guidelines, and applicable regulatory requirements, including the Declaration of Helsinki and the ICH harmonised tripartite guideline. Ethics approval was granted by the Bellberry Human Research Ethics Committee, Eastwood, South Australia (ethics approval number HREC2020‐07‐622).

Only healthy volunteers that met the specified inclusion and exclusion criteria were able to participate in this study (Table S2). After participant screening, 11 enrolled participants received two skin punch biopsy‐induced wounds, one to each upper inner arm. In each patient, in a randomised process, the wound in one arm was assigned to be the treatment wound and received a subcutaneous injection of LEAP‐isolated pEVs formulated as a clinical‐grade, allogeneic therapeutic product. The wound in the other arm was assigned to be the comparator wound and received a subcutaneous injection of a placebo formulation. Participants were evaluated for safety outcomes and skin punch biopsy wound closure.

### 2.14 Clinical trial procedure

All participants provided informed, written consent to participate in the trial after discussion with the Principal Investigator or Sub‐Investigator. Clinical pEVs, which consisted of a sterile formulation of the LEAP‐purified human non‐autologous pEV (300 μg/mL) in an isotonic solution containing sodium chloride, histidine, sucrose, polysorbate 20, and water for injection, was stored at −80°C (± 15°C). The placebo, which consisted of the same isotonic solution, minus the pEVs, was also stored at −80°C (± 15°C). On the day of dosing, the selected vials of clinical pEVs and placebo were transferred to a 2–8°C refrigerator to thaw for 1 h before administration. After removal from the storage refrigerator for administration, each vial was gently hand warmed to ambient temperature with gentle agitation to prevent foaming.

On Day 0, participants underwent physical examination, including vital signs recording (blood pressure, pulse rate, respiration rate, and temporal temperature). They received a bilateral 4 mm skin biopsy to each inner upper arm using a sterile punch following a standard clinical practice. Participants were administered a single dose (100 μg in 340 μL) of clinical pEVs injected subcutaneously adjacent to the assigned arm wound site and an equivalent volume (340 μL) of placebo injected subcutaneously to the comparator wound site. Participants and trial investigators were blinded as to which treatment had been administered to which arm. Wounds were dressed using standard of care procedures. Participants were observed in the outpatient clinic for 1 h following treatment, noting any local adverse reaction at the injection site. Participants were then discharged.

The safety of the clinical pEVs was monitored over the next 30 days. On Day 3, the site healthcare professional removed the dressing from the participant, and the wound was washed with sterile saline before the examination. The blinded follow‐up assessor(s) did not have access to participant study notes recording to which arm clinical pEVs/placebo was administered. A physical examination and vital signs check were conducted on days 3, 7, 14, and 30. The wound sites were examined for erythema, induration, exudate, fibrin coverage, wound margin, granulation issue, evidence of infection, and wound closure rate.

To further assess the safety of the intervention, the general laboratory blood examination performed during participant screening was repeated on days 7, 14, and 30 by taking blood samples for complete blood examination (red blood cells, white blood cells, haemoglobin, hematocrit, erythrocyte sedimentation rate, platelets), blood coagulation factors (activated partial thromboplastin time, prothrombin time and thrombin time) and general biochemistry for metabolites and hormones (liver function tests, C‐reactive protein, creatinine, urea, albumin, haemoglobin A1c, blood glucose, glomerular filtration rate, cholesterol and thyroid stimulating hormone).

## 3 RESULTS

### 3.1 Characterization of pEVs purified by LEAP

Platelet releasate from activated platelet packs was processed through a chromatography‐based EV isolation protocol, LEAP, to produce purified pEVs (Law et al., 2021). Each purified batch of pEVs was characterized for yield, size, concentration, morphology, and protein content.

**FIGURE 1:**
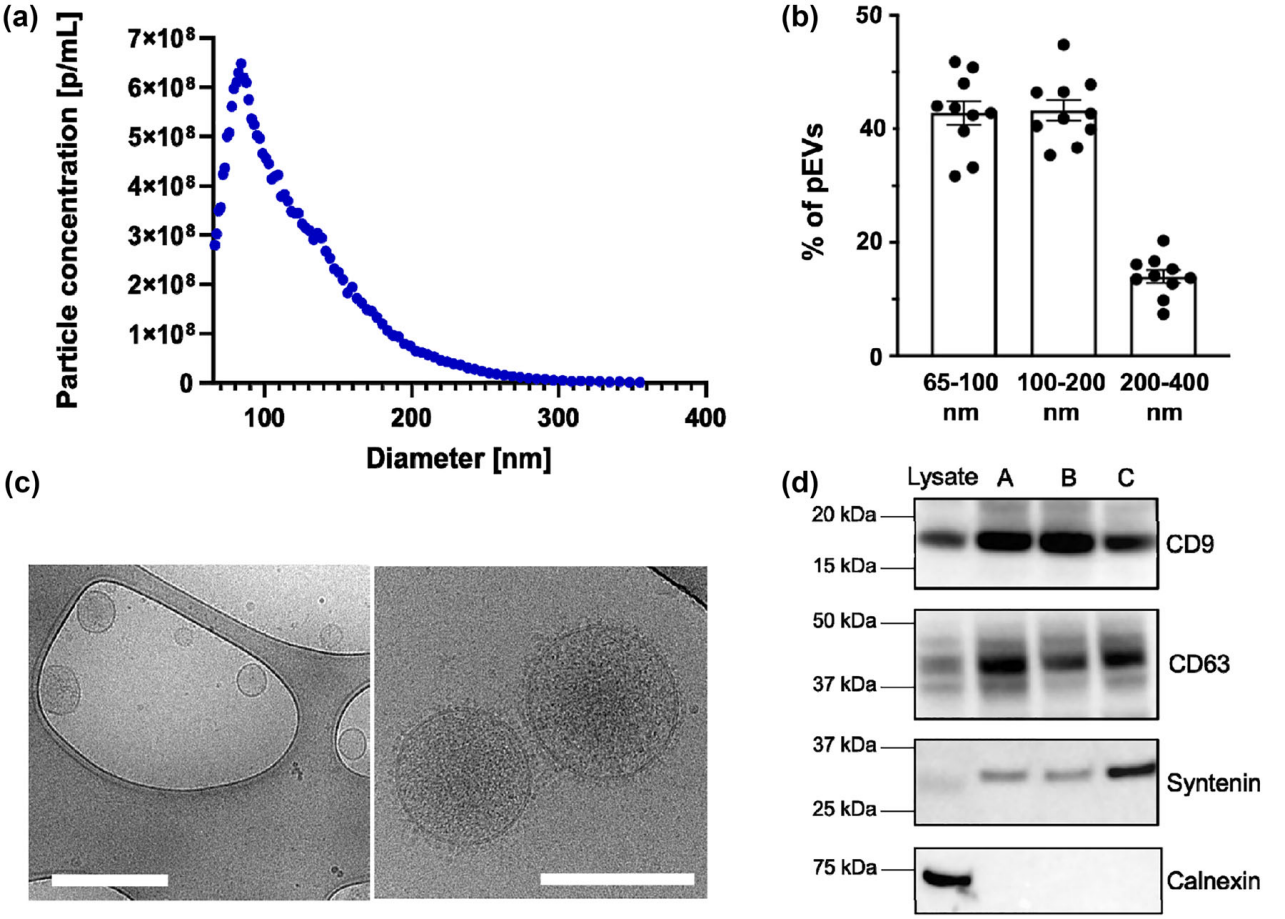
Characterization of LEAP‐purified pEVs. (a) Size distribution of clinical pEVs measured by Spectradyne, detecting pEVs from 65 to 400 nm. (b) Percentage of pEVs in clinical pEVs present within the size ranges of 65–100, 100–200, and 200–400 nm. (c) Cryo‐TEM images (left zoomed out, right zoomed in) of clinical pEVs, confirming the size distribution and innate morphology of the pEVs. Scale bar: 500 nm (left), 200 nm (right). (d) Western blot analysis of clinical pEVs showed enrichment of known EV markers CD9, CD63, and syntenin relative to platelet lysate and showed the absence of the negative EV marker, calnexin.

The product passed tests for sterility and for endotoxin (data not shown). Protein content was measured by BCA protein assay, and 1.41 ± 0.34 mg total protein was derived from each pooled platelet pack. Each vial of drug product contained 0.3 ± 0.1 mg/mL of protein and a total volume of 0.5 mL. Placebo vials were produced containing sterile filtered formulation buffer. Particle analysis was performed by Spectradyne and the total number of pEVs isolated from each pack was determined to be 8.71 ± 2.75 × 10^11^ with the expected size range of 65–400 nm (Figure 1a). Of the EV population, 42.8 ± 2.1% were small EVs between 65 amd 100 nm in size, 43.2 ± 1.8% were 100–200 nm in size, and 14 ± 1.1% were between 200 and 400 nm in size (Figure 1b).

Size distribution and consistent morphology were confirmed by cryo‐TEM (Figure 1c). As expected for EVs, the zoomed‐in image evidenced the presence of a clear lipid bilayer with protein embedded on the surface. Vesicles appear darker on the inside, indicating the presence of an electron‐dense cargo within each pEV. Western blot analysis showed enrichment of known EV markers CD9, CD63, and syntenin relative to platelet lysate, and showed the absence of the negative EV marker, calnexin (Figure 1d).

### 3.2 Characterization of pEV proteome by mass spectrometry

**FIGURE 2:**
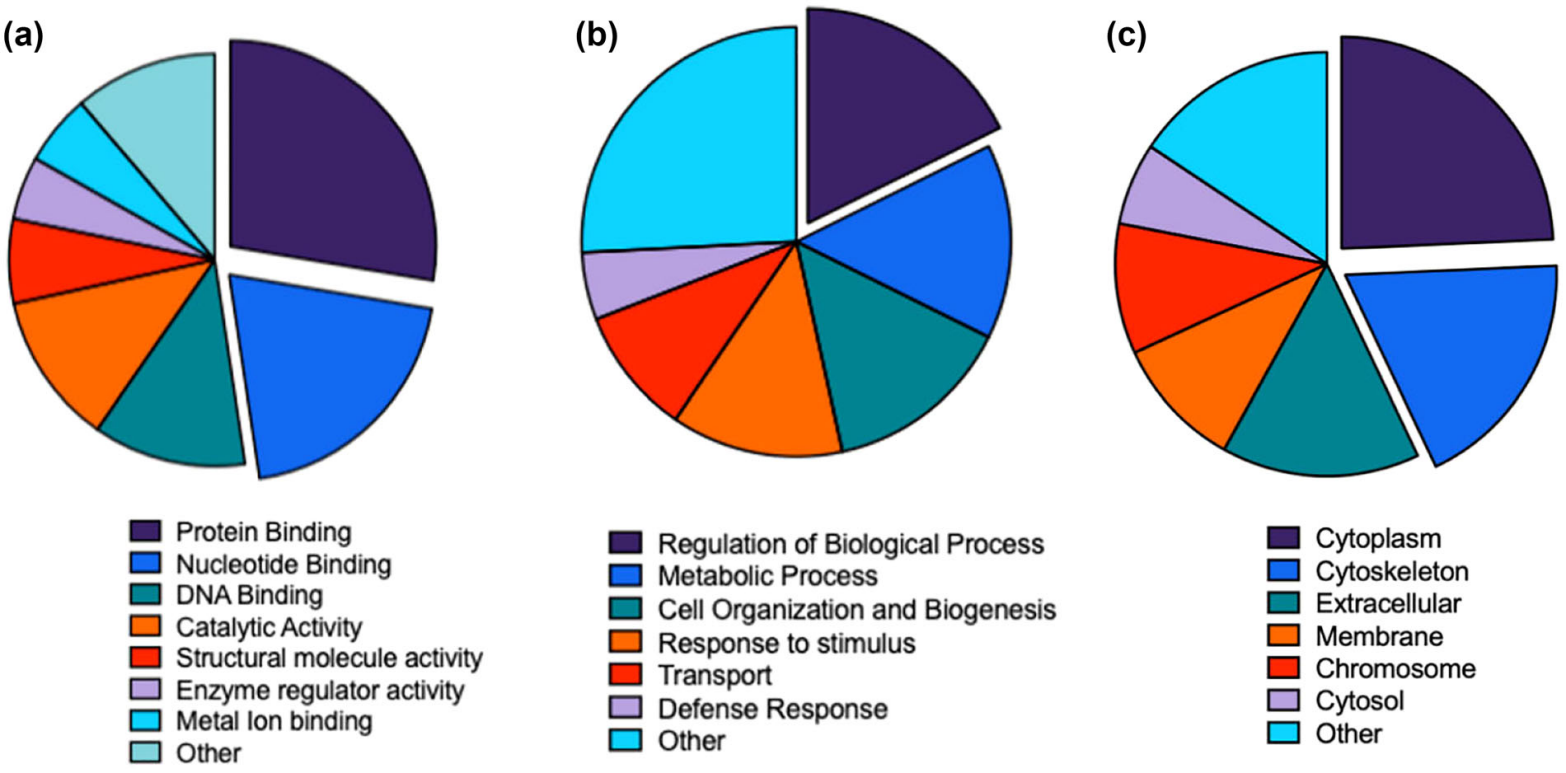
Mass spectrometric analysis revealed the abundance of proteins present in clinical pEVs based on whether they are involved in: (a) molecular function, (b) biological processes and (c) subcellular localisation.

To establish the proteome of pEVs isolated by LEAP, purified pEVs were digested and analysed by mass spectrometry. A total of 928 proteins were identified by this process, including expected EV biomarkers such as CD9, Annexins, and heat shock proteins. Extracellular vesicles generally display membrane proteins that mimic the parent cell (French et al., 2020; Fritzsching et al., 2002). In this study, pEVs were found to possess several platelet membrane proteins known to be associated with wound healing. The proteins identified included glycoproteins Ia, Ib, IV, V, VI, IX, and IIb‐IIIa, which contribute to the coagulation cascade during healthy wound healing and serve as surface receptors for binding thrombospondin and fibrinogen. Also identified were growth factors, including insulin growth factor (IGF) and transforming growth factor beta (TGF‐ß). These growth factors are known inducers of rapid re‐epithelization and known to promote the expression of myofibroblasts, thus facilitating the remodelling phase of wound healing (Baum & Arpey, 2005; Epstein et al., 1999). Proteins identified were annotated under the following categories: (A) Molecular Function, (B) Biological Processes, and (C) Sub‐cellular localization (Figure 2). Under Molecular Function, the most significant proportion of proteins was found to be protein‐binding (27.7%) and nucleotide‐binding (20.0%), as seen in Figure 2(a). A total of 32.3% of proteins identified appear to be involved in regulating biological and metabolic processes. A smaller proportion of proteins are involved in cell biogenesis (14.2%) and response to stimulus (13.0%) (Figure 2b). Sub‐cellular locations indicate that proteins from cellular cytoplasm and cytoskeleton contribute 21.0% and 16.2% of total proteins, respectively (Figure 2c).

### 3.3 Association of Exoria‐labelled pEVs with dermal fibroblasts

**FIGURE 3:**
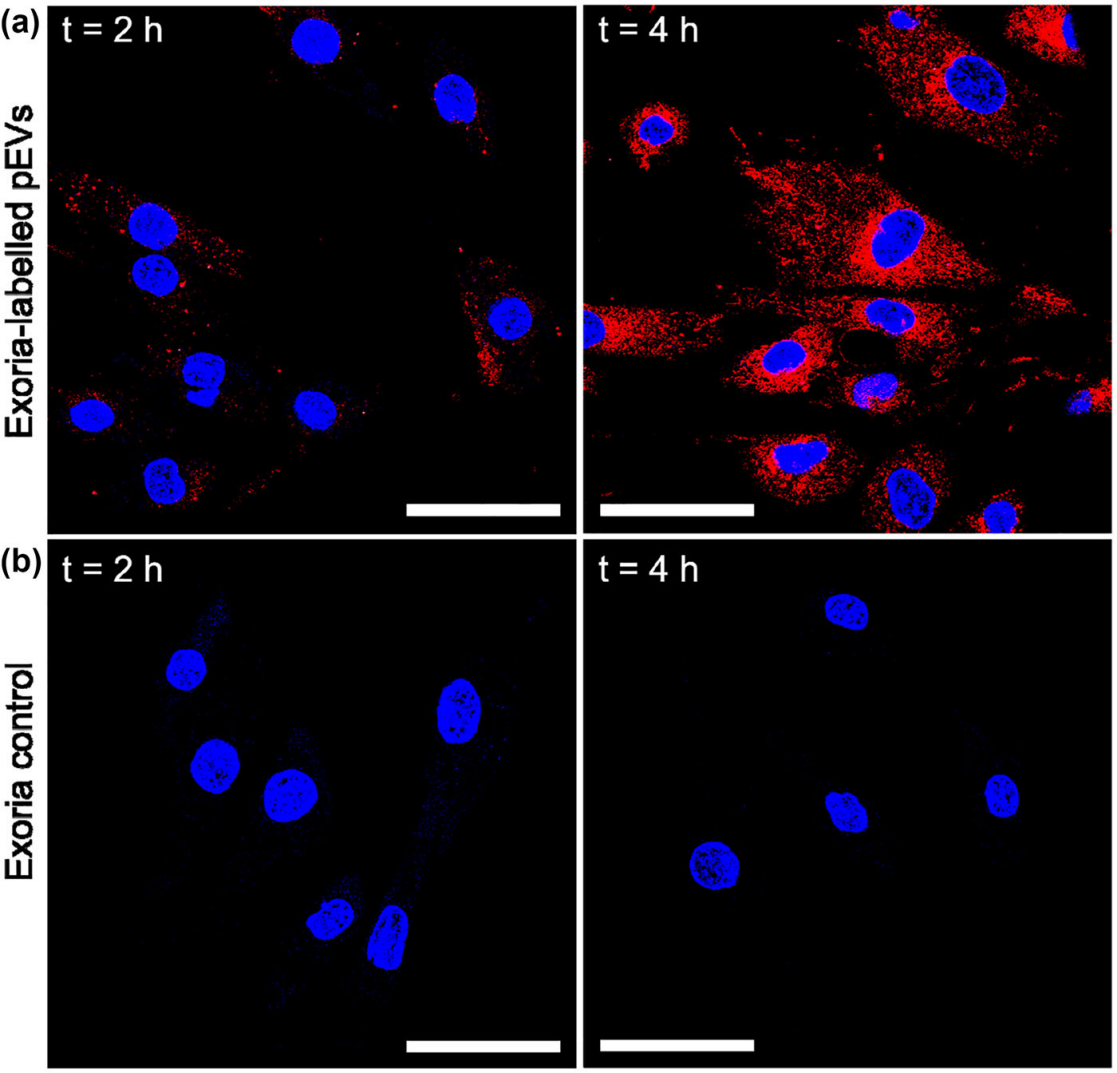
Confocal microscopy images demonstrate (a) pEV association with NHDFs, which increases over time of incubation, as opposed to (b) Exoria control, where no signal is detected. Red: Exoria‐labelled pEVs, blue: DAPI (nucleus). Scale bar: 10 μm.

A series of cell‐based studies were performed to assess the biological activity of the LEAP‐isolated pEVs for wound healing. First, to confirm that the isolated pEVs retained the ability to be taken up by relevant cells, pEVs were labelled using the lipophilic dye Exoria, which has been used to monitor cell uptake in previous reports (Tertel et al., 2022). Following 2 h of incubation, red fluorescence was detected within normal human dermal fibroblasts (NHDFs), which increased after 4 h, indicating that Exoria‐labelled pEVs are able to successfully associate with fibroblasts (Figure 3).

### 3.4 Cellular response to pEV treatment

**FIGURE 4:**
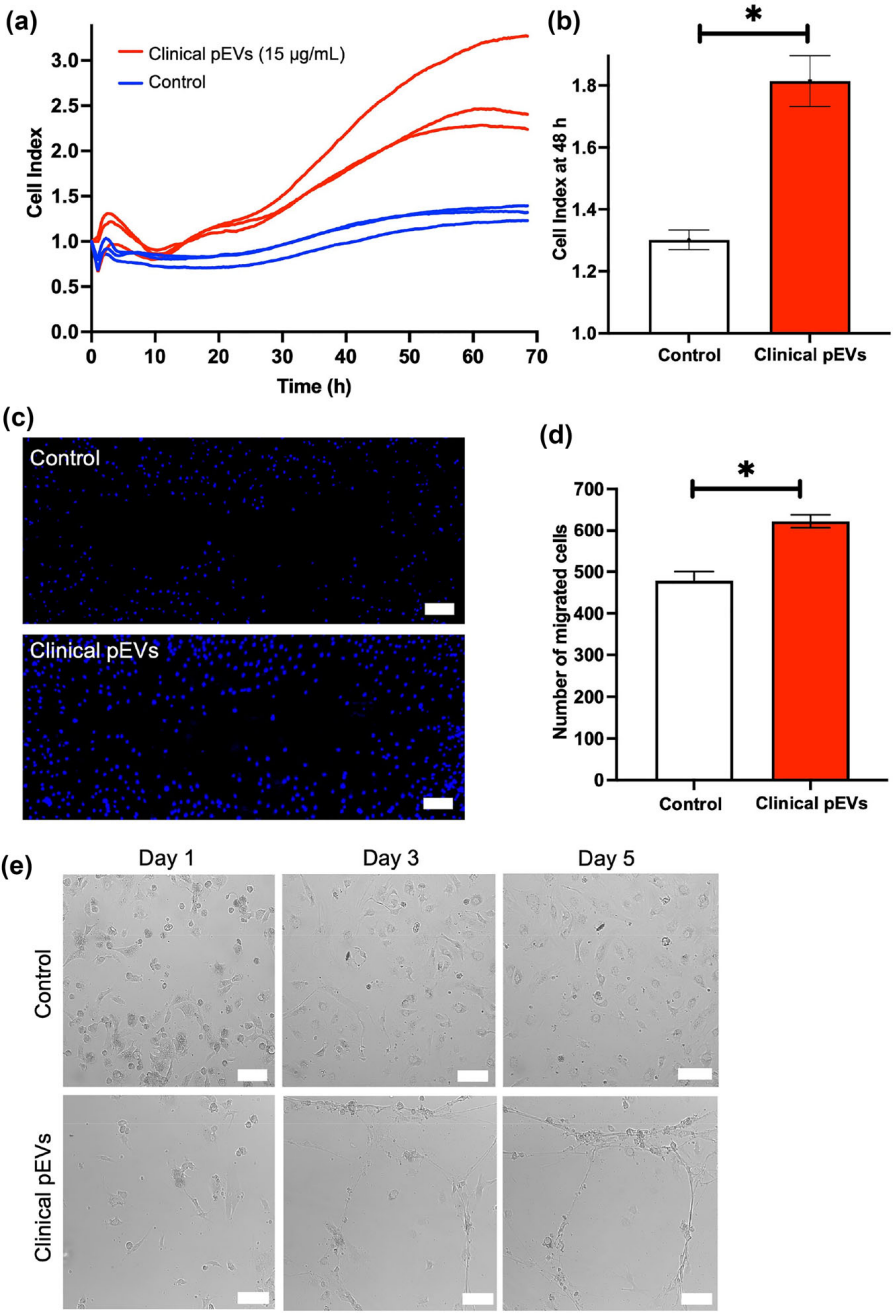
(a) Growth curve of NHDF cells in minimal growth media over 70 h treated with 15 μg/mL of three batches of clinical pEVs (red) and without any treatment (blue). (b) Cell index of clinical pEV‐treated NHDF cells versus no treated cells after 48 h of incubation, demonstrating that clinical pEVs significantly improved cell proliferation (*n* = 3, *p < 0.05, one‐way ANOVA). (c) Representative images of scratched area displaying NHDF cells migration capacity when untreated (top) or treated with clinical pEVs (bottom). Blue: DAPI (nucleus). (d) Quantification of migrated cells in treated and untreated cells after 24 h using ImageJ (*p < 0.05, one‐way ANOVA). (e) Microscope images of HDMEC cells with clinical pEVs (bottom) and without (top) clinical pEVs treatment at different time points: day 1, day 2 and day 5. After approximately day 3, clinical pEV‐treated HDMEC cells show tubular formation due to enhanced angiogenesis capacity. Scale bar: 100 μm.

Cell migration and proliferation are essential steps in regenerating injured tissue (Baum & Arpey, 2005). pEVs have been shown to increase the proliferation rate of many cell types, including fibroblasts, endothelial cells, and mesenchymal stem cells (Tao et al., 2017; Torreggiani et al., 2014). To assess this effect in cells associated with wound healing, LEAP‐purified pEVs (15 μg/mL) were added to NHDFs grown in basal growth media. pEV‐treated cells showed significantly increased proliferative capacity (cell index) (Figure 4a), particularly after 48 h (Figure 4b), relative to untreated control. Similarly, after scratching the NHDF cell monolayer, treated cells could migrate to the scratched area within 24 h post‐treatment. In contrast, a limited number of cells were visualised in the scratched area for untreated cells (Figure 4c,d).

The formation of new vasculature is a critical step in the wound healing process (Epstein et al., 1999). Tube formation of capillary‐like tube structures by endothelial cells is an established angiogenesis in vitro assay (Irina & Hynda, 2010; Kubota et al., 1988). The potential of pEVs to promote angiogenesis was assessed using human dermal microvascular endothelial cells (HDMECs). The tube formation capacity of treated cells was compared to that of untreated controls. After incubation over 5 days, HDMECs treated with pEVs formed elongated structures, suggesting subtle support of network formation, while untreated control cells retained their cobble‐stone structure, indicating that pEVs increased the angiogenic potential of endothelial cells (Figure 4e).

### 3.5 Molecular response of dermal fibroblasts to LEAP‐purified pEV treatment

**FIGURE 5:**
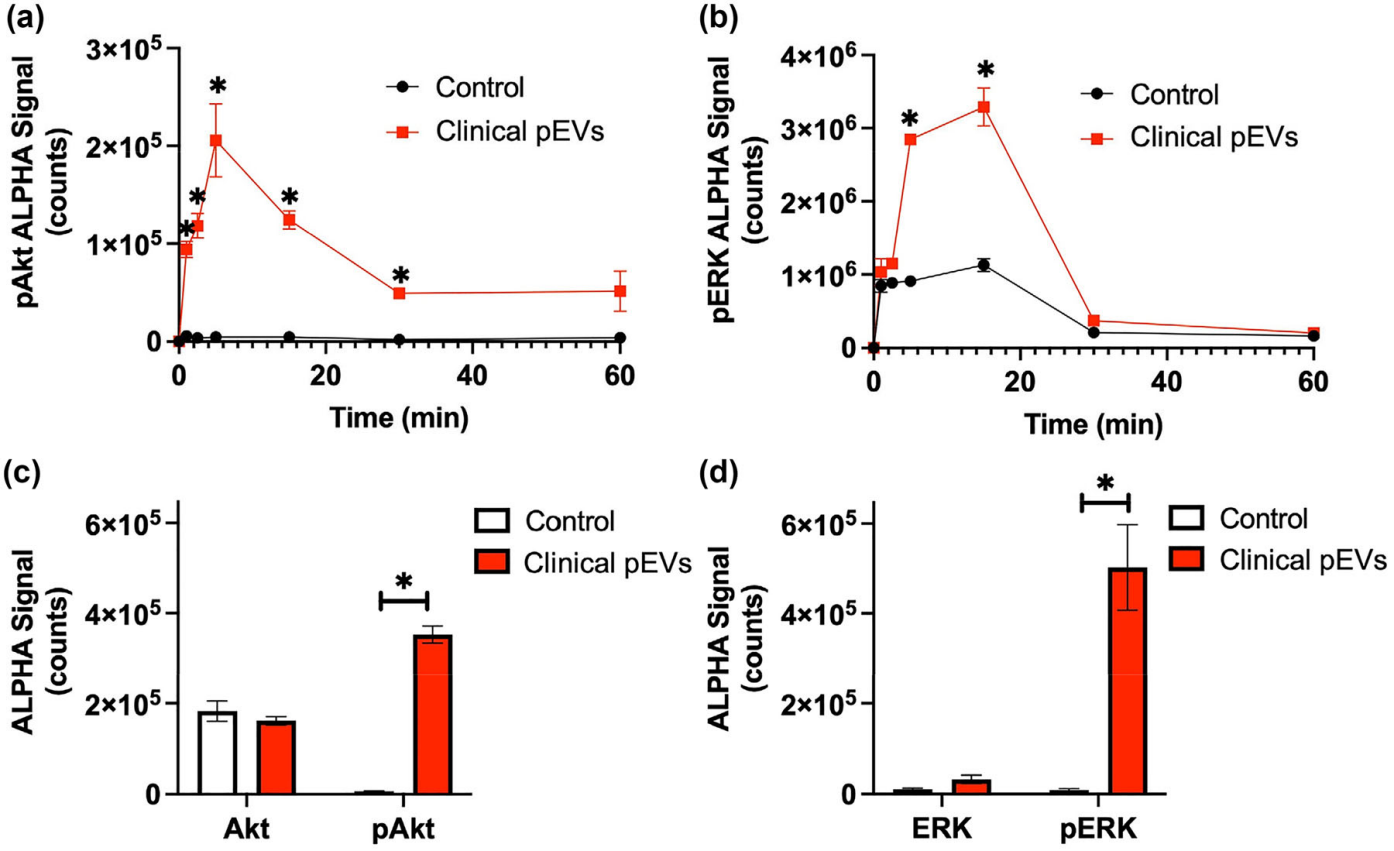
Measurement of (a) pAkt/Akt and (b) pERK/ERK ratios over time when untreated (black) or treated with pEVs (red). Comparison of (c) Akt and pAkt levels, and (d) ERK and pERK levels in cells after 5 min incubation with clinical pEVs (red) as opposed to untreated cells (white). *n* = 6, *p < 0.05, one‐way ANOVA.

The effect of pEV on vital cellular pathways was assessed to determine the mechanisms by which pEVs may induce the observed range of cellular responses in treated cells. After treatment with pEVs, AlphaLISA assays were performed to detect ERK and Akt phosphorylation in NHDFs over the course of 2 h (Figure 5). pEVs induced phosphorylation of both ERK and Akt signalling pathways, which have downstream effects on cell growth and survival. Compared to untreated cells, pAkt levels, normalised to Akt, were measured at significantly higher amounts after 1 min of incubation with pEVs, peaking at 5 min post‐treatment (Figure 5a,c). Similarly, significant differences in pERK/ERK levels were detected at 5 and 15 min post‐treatment compared to untreated cells (Figure 5b,d).

### 3.6 Safety of clinical pEVs

The safety of the clinical pEV treatment was assessed using a prospective, randomised, double‐blind, placebo‐controlled, single dose, phase I clinical trial. After receiving a skin punch biopsy wound to each inner arm, 11 participants were randomised and treated with clinical pEVs to one wound and with placebo to the comparator wound. All 11 participants completed the study (i.e., 100.0%). Most participants were male (8/11, 72.7%) and white (10/11, 90.9%), with a mean age of 29.0 years (Table S1).

No reported study deaths, serious adverse events, or adverse events leading to withdrawal of a study participant, were recorded (Table S3). No clinically meaningful abnormalities were observed in the treatment or placebo group.

**FIGURE 6:**
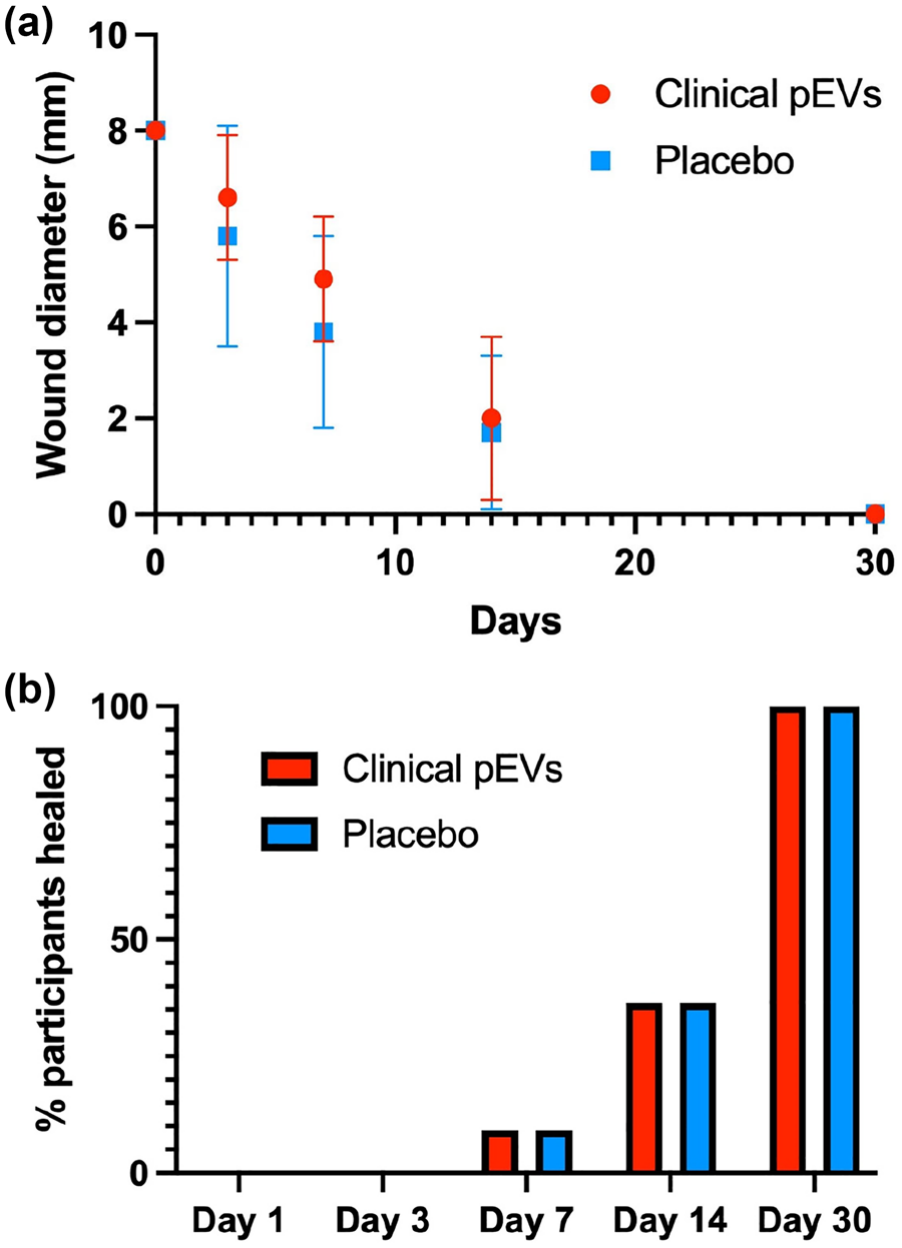
(a) Plot of wound healing rate, showing the wound diameter (defined as the sum of the vertical and horizontal diameter) of treated (red) and untreated (blue) 4 mm punch biopsy induced wounds over time. (b) Plot of wound healing rate, showing the number of treated (red) and untreated (blue) wounds to have completely healed, as recorded during participant physical examination.

Clinical pEV‐treated wounds healed successfully. A mean healing time of 22.8 ± 8.7 days was recorded for both the clinical pEV and the placebo groups. All (11 of 11 [100.0%]) participants had achieved wound closure at Day 30 of the treatment period for both treatment groups and no abnormal scar formation or other cosmetic deformities were recorded (Figure 6; Table S4).

## 4 DISCUSSION

Several preclinical studies have demonstrated that pEVs represent a promising potential wound healing therapeutic (Guo et al., 2017; Johnson et al., 2020; Torreggiani et al., 2014). A limiting factor in therapeutic EV development has been the lack of a readily scalable method for clinical‐grade EV purification (Colao et al., 2018). In this study, we purified pEVs through the scalable chromatography‐based clinical EV manufacturing protocol, LEAP (Joseph et al., 2018; Law et al., 2021). LEAP‐purified pEVs conformed to the MISEV guidelines for EV isolates, showing the expected EV biophysical characteristics and protein biomarkers (Théry et al., 2018). LEAP‐purified pEVs also demonstrated efficacy in several in vitro models of wound healing. Clinical grade pEVs manufactured through the LEAP protocol were then assessed in a phase I clinical trial for safety in the context of wound healing. No significant adverse events were reported, indicating that pEVs can safely be administered to humans and warrant further clinical trials for wound healing efficacy.

Blood and its derivatives are complex biofluids from which to isolate EVs (Clayton et al., 2019). A bottleneck in translating EVs into human use has been the lack of GMP‐compatible EV purification and manufacturing protocol amenable to scale up. Research‐scale EV purification is typically performed by ultracentrifugation (UC), which isolates EVs from impurities based on their density. However, this protocol is not directly scalable, is labour‐intensive, and leads to poor product purity (Colao et al., 2018; Lee et al., 2019). Tangential flow filtration (TFF) or size‐exclusion chromatography (SEC), in contrast, are highly scalable but can show low purity as they cannot separate EVs from contaminants of the same size (Paganini et al., 2019). TFF can be combined with SEC for enhanced purity (Watson et al., 2018) but at the expense of time and yield.

LEAP chromatography is an affinity‐like, ion‐exchange resin‐based biologic purification protocol highly amenable to manufacturing scale‐up of clinical‐grade material. Ion‐exchange resin‐based chromatography is well‐established in the commercial production of proteins and antibodies (Liu et al., 2010) and virus particles (Burden et al., 2012). Heath *et al.* reported that implementing ion‐exchange chromatography (IEX) for the purification of EVs requires much less hands‐on time than UC and produces EVs of improved quality and purity compared to those from TFF (Heath et al., 2018).

LEAP resins are IEX resins based on cation exchange. Although EVs have a net‐negative charge, local areas of positive charge exist on the outer surface of their lipid bilayer structure. LEAP technology uses charged ligand groups spaced 5 Å apart along the resin backbone to interact with those positive charges to capture EVs (Joseph et al., 2018; Law et al., 2021). The advantages of LEAP over other techniques for large‐scale EV manufacturing, in terms of yield and purity, have been previously reported and discussed (Law et al., 2021).

To validate the use of LEAP to produce clinical‐grade EVs at scale and to explore the potential of LEAP‐purified pEVs as a wound healing therapeutic, human platelet packs donated for research were sourced from a clinical‐grade platelet supplier. Pooled platelet packs were activated by an in‐house protocol to stimulate pEV release, and the pEVs were then isolated from the releasate using LEAP chromatography. The LEAP unit operation proved to be time‐efficient, does not require manual operator intervention once set up, and can be performed by two operators working approximately 8 h to purify the EVs from each activated platelet pack.

The pEVs purified through the LEAP protocol were characterised using a range of techniques and were found to display the expected EV biophysical characteristics. Proteomic analysis was performed to analyse the protein cargo of LEAP‐purified pEVs. The pEVs carried the expected EV protein markers, including CD9, annexins, and heat shock proteins (Théry et al., 2018). Previous studies have shown that pEVs can be highly enriched in proteins that support healing, including growth factors (Guo et al., 2017; Torreggiani et al., 2014). Our proteomic analysis revealed that isolated pEVs displayed the expected protein cargo, including glycoproteins Ia, Ib, IV, V, VI, IX, and IIb‐IIIa, which are platelet proteins known to play a vital role in the haemostasis phase of wound healing (Kunicki, 1989), and growth factors including IGF and TGF‐ß that have been shown to instigate the proliferative wound healing phase. Our bioinformatics analysis did not detect platelet derived growth factor (PDGF) or brain‐derived neurotrophic factor (BDNF); however, this may be because the mass spectrometric analysis was conducted in a DDA (data dependent acquisition) format. In highly complex samples, peptide species present in low abundance may not be selected for fragmentation by the instrument and so not detected by the bioinformatics software.

Fibroblasts and endothelial cells have previously been shown to increase their proliferation rate in response to pEV treatment (Guo et al., 2017; Torreggiani et al., 2014). We used a lipophilic red fluorescent dye, Exoria, to label LEAP‐isolated pEVs and showed that they could associate with dermal fibroblasts (Figure 3). In our study, dermal fibroblasts treated with pEVs showed the expected significant increase in cell proliferation and enhanced migration capacity compared to untreated cells (Figures 4 and 5), in agreement with previous work (Guo et al., 2017). Batch to batch differences in the proliferative response observed using the xCelligence assay (Figure 4a) may be attributed to the high natural biological variability among platelet donors. Typically, commercially‐produced platelet derivatives, such as platelet lysate, addresses this variability by pooling donations from at least ten and up to fifty donors (Johnson et al., 2020). Extensive pooling was not conducted for the current study due to limited supply of platelet donations for research use, but would be performed to produce Plexaris reproducibly at clinical scale.

The migration and proliferation of several cell types into the wound bed are crucial steps in the healing of wounds (Baum & Arpey, 2005). The transition from the inflammatory to the proliferative phase of wound healing is characterised by the rapid proliferation of endothelial cells and dermal fibroblasts. Activated fibroblasts deposit extracellular matrix components such as immature type III collagen, which is essential for reepithelialisation and wound closure (Epstein et al., 1999). Chronic wounds typically fail to progress from the inflammatory to the proliferative phase of wound healing; this cell type is particularly interesting for reactivating stalled healing. Our observations that pEVs can stimulate fibroblast proliferation and migration suggest that pEV treatment can potentially drive stalled wounds toward healing.

Angiogenesis is a critical facet of wound healing. New capillary formation supplies the wound with the required oxygen and nutrients to support recovery. Impaired circulation and poor angiogenesis are known risk factors in, for example, non‐healing diabetic ulcers (Prompers et al., 2008). pEVs can promote angiogenesis (Guo et al., 2017; Torreggiani et al., 2014). In agreement with previous work, endothelial cells treated with LEAP‐purified pEVs spontaneously formed tube‐like structures, which then connected to form networks, indicating the initiation of new capillary development (Figure 4) (Guo et al., 2017). No morphological change toward new blood vessel generation was observed in untreated control cells.

To probe the LEAP‐purified pEVs’ possible cellular mode of action, activation of the ERK and Akt signalling pathways in dermal fibroblasts was assessed. The ERK pathway drives the proliferation and migration of multiple cell types, including keratinocytes and epithelial cells (Lee et al., 2018). Growth factors, including epidermal growth factor (EGF) and platelet‐derived growth factor (PDGF), have been shown to activate the ERK pathway (Balli et al., 2020; He et al., 2012; Matsubayashi et al., 2004). Similarly, the Akt pathway modulates several downstream functions, such as proliferation and cell survival, and Akt and ERK signalling has been shown to play a role in wound healing (Huang et al., 2015; Lee et al., 2018). Downregulated ERK and Akt pathways have been implicated in the pathogenesis and progression of chronic wounds (Achar et al., 2014; Kim et al., 2003). In the present study, we showed that applying pEVs to dermal fibroblasts induced a three‐fold increase in phosphorylated ERK and Akt within treated cells 5 min after treatment, indicating the activation of both pathways. ERK and Akt phosphorylation of endothelial cells was also noted by Guo et al. 30 min after pEV treatment (Guo et al., 2017). Phosphorylation at earlier time points was not analysed. These findings suggest that pEVs could activate key cellular pathways associated with wound healing.

Studies conducted to date suggest that EV therapeutics have a strong safety profile. The thousands of blood and plasma transfusions conducted daily, in which non‐autologous EVs are safely transferred into patients in large numbers, is one positive indicator of EV safety. EVs have also been tested in a small but growing number of early‐stage human clinical trials, and shown to be generally well tolerated (Kordelas et al., 2014; Nassar et al., 2016; Perocheau et al., 2021; Xiaomin et al., 2018). Having demonstrated the bioactivity of LEAP‐purified pEVs by in vitro assays representing aspects of wound healing, we initiated a phase I human clinical trial to assess the safety of our product as a future wound healing treatment (Plexoval II study, ACTRN12620000944932). To assess the safety of clinical pEVs in healthy individuals in the context of wound healing, eleven healthy volunteers meeting the specified criteria for participation received a 4 mm skin punch biopsy to each inner upper arm. In each volunteer, one arm was randomly assigned to receive the pEV therapeutic, and the other received a placebo injection. No deaths, serious adverse events, or adverse events leading to withdrawal of a study participant, were reported. LEAP‐purified pEVs were found to be safe when administered to humans, meeting the primary endpoint of the clinical trial.

The secondary objective of the clinical study was to conduct an exploratory assessment of the bioactivity of the pEV treatment. No difference was observed in the total time to wound closure between the treated and untreated arms. A mean healing time of 22.8 ± 8.7 days was recorded for both groups, and each wound had entirely healed within 30 days. There was no difference in the physician wound evaluation between treatment groups.

As a phase I study focused on assessing safety, several aspects of the study design may explain the lack of observed bioactivity in this clinical trial. Based on previous studies and our in vitro assays, delayed wound healing is the target indication of the LEAP‐purified pEV product. However, the delayed healing patient cohort is typically elderly, and displays health complexities including multiple co‐morbidities such as diabetes, cardiovascular disease, obesity and peripheral vascular disease. Therefore, as a first‐in‐human study, a trial design based on assessing product safety in a small number of healthy participants was selected. The trial design was not powered to draw conclusions about therapeutic efficacy. As such, in depth analysis of product bioactivity, such as wound histology during healing, was not performed.

Although an exploratory assessment of wound healing rate was conducted, a 4 mm skin punch biopsy wound in a healthy individual naturally heals rapidly, making any differences in healing time between the clinical pEV treatment and placebo challenging to observe. Furthermore, wound healing from a skin punch biopsy in a healthy individual differs significantly from healing in a chronic wound in a patient with an underlying health condition, such as a non‐healing diabetic ulcer. A healthy wound is not expected to be deficient in the biomolecules that pEVs carry, which may further explain the lack of bioactivity observed. Conversely, patients with chronic wounds suffer from inhibition of the biological events that lead to healing, which pEVs could be able to reverse. For example, in a previous animal study, treatment with pEVs leads to faster wound healing in a rat model of diabetic ulcer than in an untreated wound (Guo et al., 2017). A further difference between the studies was that the rat wounds were treated with a pEV‐loaded hydrogel that eluted the therapeutic over several days rather than treated with a single injection. These differences should be considered in future clinical trials, designed to test pEV efficacy in a defined delayed wound healing patient cohort.

In conclusion, we have shown proof of concept that LEAP chromatography is amenable to the scalable manufacture of clinical grade pEVs from platelet cell releasate as an experimental therapy for wound healing. The pEVs were shown to be active in a range of wound‐healing in vitro assays and safe when administered to healthy volunteers in a phase I clinical trial for safety in the context of wound healing. All wounds healed rapidly and completely and no difference in time to wound closure of the treated and untreated wounds was observed. Although the secondary endpoint of the trial, to assess efficacy, was not met in this cohort of healthy volunteers at this dose, the result was not unexpected in the context of a trial designed to assess safety involving only healthy volunteers with a normal swift wound healing response. Further exploration of the efficacy of the pEV therapeutic in future clinical trials for delayed wound healing would be warranted.

## CONFLICT OF INTEREST STATEMENT

All authors are or were employed or received financial support from Exopharm Ltd. Jancy Johnson, Sam Q. K. Law, Sadman Bhuiyan, Anabel Silva, Melanie Schoppet, Chantelle Blyth, Owen C. Tatford, Anna Cifuentes‐Rius, Patrick F. James, Angus Tester, Ian Dixon, Gregor Lichtfuss are shareholders of Exopharm Ltd.

## Supporting information

Supporting Information
