# Supplementary material for: First‐in‐human clinical trial of allogeneic, platelet‐derived extracellular vesicles as a potential therapeutic for delayed wound healing
Source: J Extracell Vesicles. 2023 Jun 23;12(7):12332. doi: 10.1002/jev2.12332 (PMC10290200; doi:10.1002/jev2.12332)
Supplement: Supplementary file 1 — Supporting Information [file JEV2-12-12332-s001.docx]

**Supplementary Information**

Table S1. Demographics and baseline characteristics of participants

|  |  | Percentage participants (%) |
| --- | --- | --- |
| Gender | Male | 72.7 |
|  | Female | 27.3 |
| Race | White | 90.9 |
|  | Asian | 9.1 |
| Ethnicity | Not Hispanic or Latino | 100 |
|  |  |  |
|  |  | Years |
| Age | Mean ± SD | 29.0 ± 10.4 |

Table S2. Plexoval II participant selection criteria

| Study Inclusion Criteria |
| --- |
| Healthy adults aged 18 to 64 years of age |
|  |
| Able to read, understand and sign Participant Information and Consent Form |
|  |
| Eligible for punch biopsy |
|  |
| Suitable health status to participate in the study, determined by medical history, vital signs, |
| physical examination and within acceptable test range for general haematological laboratory |
| profiles including full blood examination (i.e. RBC, WBC, Hb, HCT, ESR & Platelets), blood |
| coagulation factors (i.e. APTT, PT & TT) and general biochemistry laboratory profiles for metabolism & hormones (i.e. LFT, CRP, creatinine, urea, albumin, Blood Glucose, GFR, HbA1c, |
| cholesterol & TSH). |
|  |
| No history of diabetes or cardiac disease (e.g. hypertension, arrhythmia, etc) which can |
| impede and delay wound healing. |
|  |
| No history of any bleeding or coagulation disorders. |
|  |
| No history of malignancy, except for fully excised Squamous Cell Carcinoma and Basal Cell Carcinoma. |
|  |
| No active or chronic diseases/disorders requiring systemic treatment, no history of |
| hospitalisation for illness within the six months prior to enrolment into study, and no major |
| surgery within the 6 months prior to enrolment into study |
|  |
| Females of child-bearing potential must be non-pregnant, (as confirmed by β-HCG |
| serum test at screening visit and urine test at dosing visit 1) and non-lactating. |
|  |
| All females of childbearing potential, and male participants with female partners of child- |
| bearing potential, must agree to take medically acceptable contraception measures whilst |
| on the treatment and for 30 days following treatment. |
|  |
| Agree to maintain wound dressings as per study instructions. |
|  |
| Study Exclusion Criteria |
| Existing scars or evidence of infection in study area (i.e. either of the upper inner arms). |
|  |
| Evidence of existing chronic dermatological conditions such as psoriasis and eczema. |
|  |
| Participation in another clinical study currently or prior participation within 3 months of first |
| visit. |
|  |
| History of any clinically important severe allergic or anaphylactic reaction or known or |
| suspected hypersensitivity to compounds similar to the Investigational Product. |
|  |
| History of any clinically important or uncontrolled systemic disease or condition as |
| determined by the Principal Investigator. |
|  |
| Concurrent administration of NSAIDs, Immunosuppressive agents, anticoagulation therapy |
| including low dose aspirin or other anti-platelet therapy, or systemic corticosteroids therapy |

Table S3. Adverse events registered during the study with 11 participants and over 30 days

| Adverse Event (AE) | Event Severity | Number of Events | Number of participants with event [% (n/N)] | Related to the study procedure | Related to investigational product |
| --- | --- | --- | --- | --- | --- |
| Cyclical neutropenia | Moderate | 1 | 9.1% (1/11) | No | No |
| Dysmenorrhea | Mild | 1 | 9.1% (1/11) | No | No |
| Hay fever | Mild | 1 | 9.1% (1/11) | No | No |
| Intermittent Headaches | Mild | 1 | 9.1% (1/11) | No | No |
| Leukopaenia | Mild | 1 | 9.1% (1/11) | No | No |
| Placebo Arm Erythema | Mild | 1 | 9.1% (1/11) | Yes | No |
| Placebo Arm Exudate | Mild | 1 | 9.1% (1/11) | Yes | No |
| Placebo Arm Tenderness | Mild | 2 | 18.2% (2/11) | Yes | No |
| Thrombocytopaenia | Mild | 1 | 9.1% (1/11) | Yes | No |
| Treatment Arm Erythema | Mild | 4 | 36.4% (4/11) | Yes | No |
| Treatment Arm Exudate | Mild | 2 | 18.2% (2/11) | Yes | No |
| Treatment Arm Oedema | Mild | 1 | 9.1% (1/11) | Yes | No |
| Treatment Arm Pain | Mild | 1 | 9.1% (1/11) | No | No |
| Treatment Arm Tenderness | Mild | 2 | 18.2% (2/11) | Yes | No |

Table S4: Wound healing over time, by wound size

| **Subject Number** | **Visit** | **Arm Side** | **Treatment / Placebo** | **Vertical diameter (mm)** | **Horizontal diameter (mm)** |
| --- | --- | --- | --- | --- | --- |
| 101 | V2: Day 3 | Left Arm | Placebo | 4 | 4 |
| 101 | V2: Day 3 | Right Arm | Treatment | 4 | 4 |
| 101 | V3: Day 7 | Left Arm | Placebo | 2 | 3 |
| 101 | V3: Day 7 | Right Arm | Treatment | 1 | 2 |
| 101 | V4: Day 14 | Left Arm | Placebo | 2 | 2 |
| 101 | V4: Day 14 | Right Arm | Treatment | 1 | 1 |
| 101 | V5: Day 30 | Left Arm | Placebo | 0 | 0 |
| 101 | V5: Day 30 | Right Arm | Treatment | 0 | 0 |
| 111 | V2: Day 3 | Left Arm | Treatment | 4 | 3 |
| 111 | V2: Day 3 | Right Arm | Placebo | 3 | 4 |
| 111 | V3: Day 7 | Left Arm | Treatment | 3 | 3 |
| 111 | V3: Day 7 | Right Arm | Placebo | 2 | 1 |
| 111 | V4: Day 14 | Left Arm | Treatment | 2 | 1 |
| 111 | V4: Day 14 | Right Arm | Placebo | 0.5 | 0.5 |
| 111 | V5: Day 30 | Left Arm | Treatment | 0 | 0 |
| 111 | V5: Day 30 | Right Arm | Placebo | 0 | 0 |
| 116 | V2: Day 3 | Left Arm | Placebo | 1.5 | 2 |
| 116 | V2: Day 3 | Right Arm | Treatment | 4 | 3 |
| 116 | V3: Day 7 | Left Arm | Placebo | 2 | 2 |
| 116 | V3: Day 7 | Right Arm | Treatment | 3 | 2 |
| 116 | V4: Day 14 | Left Arm | Placebo | 1 | 1 |
| 116 | V4: Day 14 | Right Arm | Treatment | 2 | 1 |
| 116 | V5: Day 30 | Left Arm | Placebo | 0 | 0 |
| 116 | V5: Day 30 | Right Arm | Treatment | 0 | 0 |
| 118 | V2: Day 3 | Left Arm | Treatment | 2 | 3 |
| 118 | V2: Day 3 | Right Arm | Placebo | 1 | 1 |
| 118 | V3: Day 7 | Left Arm | Treatment | 2 | 2 |
| 118 | V3: Day 7 | Right Arm | Placebo | 0 | 0 |
| 118 | V4: Day 14 | Left Arm | Treatment | 0 | 0 |
| 118 | V4: Day 14 | Right Arm | Placebo | 0 | 0 |
| 118 | V5: Day 30 | Left Arm | Treatment | 0 | 0 |
| 118 | V5: Day 30 | Right Arm | Placebo | 0 | 0 |
| 119 | V2: Day 3 | Left Arm | Treatment | 4 | 3 |
| 119 | V2: Day 3 | Right Arm | Placebo | 4 | 4 |
| 119 | V3: Day 7 | Left Arm | Treatment | 2 | 3 |
| 119 | V3: Day 7 | Right Arm | Placebo | 3 | 3 |
| 119 | V4: Day 14 | Left Arm | Treatment | 0.5 | 1 |
| 119 | V4: Day 14 | Right Arm | Placebo | 0.2 | 0.2 |
| 119 | V5: Day 30 | Left Arm | Treatment | 0 | 0 |
| 119 | V5: Day 30 | Right Arm | Placebo | 0 | 0 |
| 120 | V2: Day 3 | Left Arm | Treatment | 3 | 4 |
| 120 | V2: Day 3 | Right Arm | Placebo | 2 | 4 |
| 120 | V3: Day 7 | Left Arm | Treatment | 3 | 3 |
| 120 | V3: Day 7 | Right Arm | Placebo | 2 | 3 |
| 120 | V4: Day 14 | Left Arm | Treatment | 1 | 1 |
| 120 | V4: Day 14 | Right Arm | Placebo | 1 | 1 |
| 120 | V5: Day 30 | Left Arm | Treatment | 0 | 0 |
| 120 | V5: Day 30 | Right Arm | Placebo | 0 | 0 |
| 123 | V2: Day 3 | Left Arm | Treatment | 3 | 3 |
| 123 | V2: Day 3 | Right Arm | Placebo | 3 | 2 |
| 123 | V3: Day 7 | Left Arm | Treatment | 2 | 1 |
| 123 | V3: Day 7 | Right Arm | Placebo | 3 | 3 |
| 123 | V4: Day 14 | Left Arm | Treatment | 0.5 | 0.5 |
| 123 | V4: Day 14 | Right Arm | Placebo | 1.5 | 1 |
| 123 | V5: Day 30 | Left Arm | Treatment | 0 | 0 |
| 123 | V5: Day 30 | Right Arm | Placebo | 0 | 0 |
| 124 | V2: Day 3 | Left Arm | Placebo | 1.5 | 1.5 |
| 124 | V2: Day 3 | Right Arm | Treatment | 2 | 2 |
| 124 | V3: Day 7 | Left Arm | Placebo | 1 | 1 |
| 124 | V3: Day 7 | Right Arm | Treatment | 2.5 | 2.5 |
| 124 | V4: Day 14 | Left Arm | Placebo | 0.2 | 0.2 |
| 124 | V4: Day 14 | Right Arm | Treatment | 0.5 | 0.5 |
| 124 | V5: Day 30 | Left Arm | Placebo | 0 | 0 |
| 124 | V5: Day 30 | Right Arm | Treatment | 0 | 0 |
| 125 | V2: Day 3 | Left Arm | Treatment | 3 | 4 |
| 125 | V2: Day 3 | Right Arm | Placebo | 3 | 4 |
| 125 | V3: Day 7 | Left Arm | Treatment | 1 | 3 |
| 125 | V3: Day 7 | Right Arm | Placebo | 1 | 1 |
| 125 | V4: Day 14 | Left Arm | Treatment | 0 | 0 |
| 125 | V4: Day 14 | Right Arm | Placebo | 0 | 0 |
| 125 | V5: Day 30 | Left Arm | Treatment | 0 | 0 |
| 125 | V5: Day 30 | Right Arm | Placebo | 0 | 0 |
| 128 | V2: Day 3 | Left Arm | Placebo | 4 | 4 |
| 128 | V2: Day 3 | Right Arm | Treatment | 4 | 4 |
| 128 | V3: Day 7 | Left Arm | Placebo | 2 | 1 |
| 128 | V3: Day 7 | Right Arm | Treatment | 3 | 3 |
| 128 | V4: Day 14 | Left Arm | Placebo | 2 | 3 |
| 128 | V4: Day 14 | Right Arm | Treatment | 1 | 1 |
| 128 | V5: Day 30 | Left Arm | Placebo | 0 | 0 |
| 128 | V5: Day 30 | Right Arm | Treatment | 0 | 0 |
| 129 | V3: Day 7 | Left Arm | Treatment | 4 | 3 |
| 129 | V3: Day 7 | Right Arm | Placebo | 3 | 3 |
| 129 | V4: Day 14 | Left Arm | Treatment | 3 | 3 |
| 129 | V4: Day 14 | Right Arm | Placebo | 0.5 | 0.5 |
| 129 | V5: Day 30 | Left Arm | Treatment | 0 | 0 |
| 129 | V5: Day 30 | Right Arm | Placebo | 0 | 0 |

Figure S1.
